# Interfacial engineering of graphene for highly efficient blue and white organic light-emitting devices

**DOI:** 10.1038/s41598-018-26464-8

**Published:** 2018-05-25

**Authors:** Shufen Chen, Qin Zhang, Wenjuan Shang, Lihui Liu, Hongtao Yu, Shuai Zhang, Lingling Deng, Min Wang, Minghao Wang, Xue Li, Baoxiu Mi, Wei Huang

**Affiliations:** 1 Key Laboratory for Organic Electronics and Information Displays & Jiangsu Key Laboratory for Biosensors, Institute of Advanced Materials (IAM), Jiangsu National Synergetic Innovation Center for Advanced Materials (SICAM), Nanjing University of Posts & Telecommunications, 9 Wenyuan Road, Nanjing, 210023 China; 2 Mechanical Engineering Institute, Nanjing Institute of Technology, Nanjing, 211167 China; 3 Institute of Flexible Electronics (SIFE), Northwestern Polytechnical University (NPU), 127 West Youyi Road, Xi’an, 710072 Shaanxi China

## Abstract

Graphene as anodes of flexible organic light-emitting devices (OLEDs) has intrinsic drawbacks of a low work function and a high sheet resistance although it can eliminate the brittle feature of ITO. Chemical doping as a conventional approach is universally used to decrease the sheet resistance and adjust the work function of graphene electrodes, but it suffers from instability problems due to the volatility of chemical species. Here, an insulated poly(4-styrenesulphonate) (PSS) modification layer is firstly coated on the graphene surface along with improved air-stability and hole-injection ability via interfacial dipoles. Besides, the utilization of PSS is beneficial to reduce the leakage current of OLEDs. Then a gradient injection layer of poly(3,4-ethylenedioxythiophene):PSS (PEDOT:PSS)/tetrafluoroethyleneperfluoro-3,6-dioxa-4-methyl-7-octenesulphonic acid copolymer-doped PEDOT:PSS is covered onto the PSS-modified graphene to further promote hole injection and improve carrier balance inside OLEDs. With above interfacial modification technique, very high efficiencies of 201.9 cd A^−1^ (76.1 lm W^−1^, 45.2%) and 326.5 cd A^−1^ (128.2 lm W^−1^, 99.5%) for blue and white emissions are obtained, which are comparable to the most efficient display and lighting technologies so far.

## Introduction

Intrinsic high transparency, remarkable conductivity and wonderful mechanical compliance made graphene an attractive electrode material for applications in flexible optoelectronic and electronic devices, e.g., organic light-emitting devices (OLEDs). The ultimate challenge in developing highly efficient OLEDs with graphene electrodes is universally considered to reduce sheet resistance and tune work function of graphene so as to realize a fine control over the carrier injection and transport^1–3^. Usually, both low sheet resistances and high work functions of graphene were achieved via chemical doping with various acids^1,4–6^, metal halides^7–13^, metal oxides^14–16^ and other organic molecules (e.g., bis(trifluoromethanesulfonyl)amide^2^ or triethyloxonium hexachloroantimonate^17^). With these chemical treatments, high luminous efficiencies of both green and white emissions over 250 cd A^−1^ (>160 lm W^−1^) and 120 cd A^−1^ (~90 lm W^−1^) were achieved in graphene-based OLEDs^17^, which are superior to device performances using ITO^18^, carbon nanotubes^19,20^, metal nanowires^21^, metal grid^22^ and other conductive polymer^23^ anodes. However, some conventional chemical dopants also induce instability problems of graphene and their devices, e.g., gradual decrease of electrical conductivity of graphene due to the volatility of acid^24^ or an increased leakage current due to large metal nanoparticles (Nanoparticles reduced from metal halides usually have sizes of 50–100 nm)^25^. In addition to a direct chemical treatment to graphene, extensive research on hole-injection layer (HIL) has been carried out to improve hole’s injection and thus balance carriers in the OLEDs with graphene electrodes. A typical example is doping tetrafluoroethyleneperfluoro-3,6-dioxa-4-methyl-7-octenesulphonic acid copolymer (PFI) into the poly(3,4-ethylenedioxythiophene):poly(4-styrenesulphonate) (PEDOT:PSS) with a volume ratio of 1:1, which can significantly enhance holes injection by increasing the graphene’s work function to a high level of 5.95 eV^1^. Recently, a sandwiched TiO_2_/graphene/PEDOT:PSS: PFI electrode architecture was used to enhance electroluminescence performance by utilizing cavity resonance effect, yielding an ultrahigh power efficiency of 250 lm W^−1^ (160 lm W^−1^ without a half-ball lens) for green emission^26^. Unfortunately, this approach is more suitable for design and development of monochromatic OLEDs instead of white ones, due to optical microcavity can only selectively enhance the emission at a certain wavelength by constraining the emissions of other wavelengths^27^.

Herein, we provide with a useful interfacial modification material poly(4-styrenesulphonate) (PSS), which not only reduces hole injection barrier by forming an interfacial dipole on graphene, but also maintains a high optical transmittance (~97% at 550 nm) and a good air-stability of the pristine single-layer graphene (SLG). With a further combination of PSS with a PEDOT:PSS/PFI-doped PEDOT:PSS gradient injection layer to sufficiently reduce hole-injection barriers, we notably demonstrate highly efficient white and blue emissions with current (power) efficiencies of 326.5 cd A^−1^ (128.2 lm W^−1^ at 5,270 cd m^−2^) and 201.9 cd A^−1^ (76.1 lm W^−1^ at 1,494 cd m^−2^) in spite of manufacturing devices on moderately conductive SLG anodes (~600 Ω sq^−1^). This study also indicates that the demanding requirements for the conductivity of graphene can be properly reduced by utilizing a gradient HIL and suitable surface modification, suggesting that a potential large-scale application of graphene in large-size OLED panels or lighting.

## Results

### PSS interfacial modification layer

**Figure 1:**
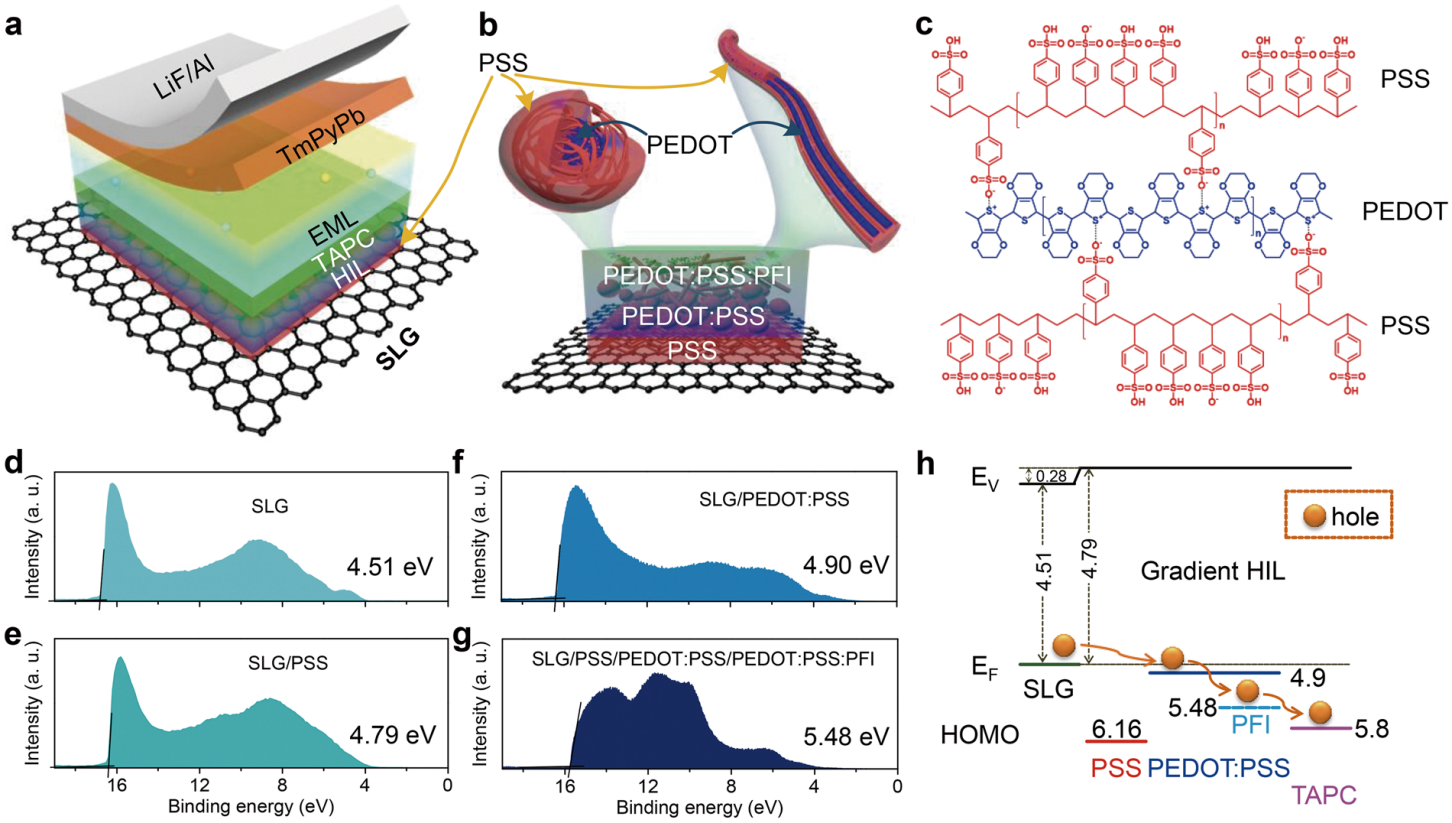
(**a**) Schematic configuration of white OLEDs. (**b**) Schematic diagram of gradient HIL including PSS, PEDOT:PSS and PFI-doped PEDOT:PSS. Part of the PEDOT:PSS grains (top left) is reformed into nanofibrils (top right) after PFI doping. (**c**) Chemical bonding of PSS with PEDOT:PSS. (**d**–**g**) He I UPS spectra of graphene, PSS, PEDOT:PSS and PFI-doped PEDOT:PSS film samples. The calculated E_F_ of graphene and PSS and HOMO levels of PEDOT:PSS and PFI-doped PEDOT:PSS are 4.51, 4.79, 4.90 and 5.48 eV. (**h**) Energy levels of gradient HIL.

The transfer approach and cleaning process of the SLG sheets used in this work are described in detail in Methods Section. Then the white OLEDs with blue and yellow complementary colors, as the schematic structure illustrated in Fig. 1a–c, are deposited onto SLG sheets via spin-coating or thermal deposition technology. In consideration of the intrinsic hydrophobicity of graphene, the as-transferred SLG is treated with UV O_3_ (6 min) prior to formation of HIL. Then the PSS aqueous solution with an optimal concentration of 3 × 10^−6^ wt% is spin-coated onto the SLG at the rotation rate of 3,000 rpm for 60 s, forming an ultrathin modification film of 2.1 ± 0.2 nm (Film thickness calculation see Methods Section). Subsequently, PEDOT:PSS and PFI-doped PEDOT:PSS are spin-coated onto the PSS modified graphene surface to comprise the HIL structure proposed in this work.

### Interfacial dipole

**Figure 2:**
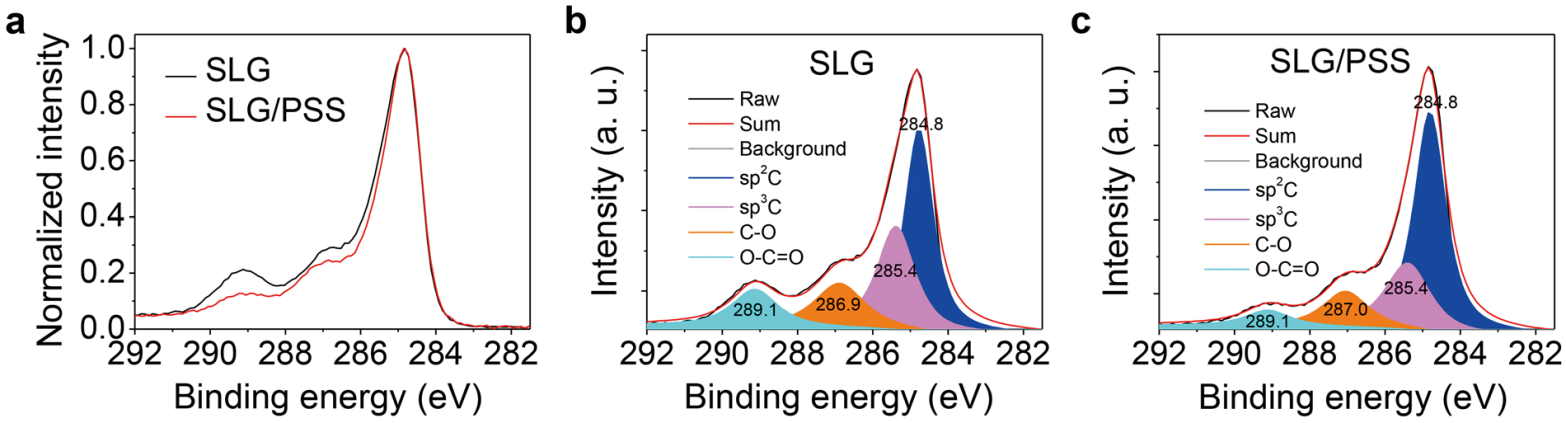
(**a**) Normalized C1s spectra of graphene sheets with and without PSS modification. The XPS C1s spectra comprised of *sp*^2^C, *sp*^3^C, C-O and O-C=O for (**b**) pristine and (**c**) PSS-modified graphene.

**Figure 3:**
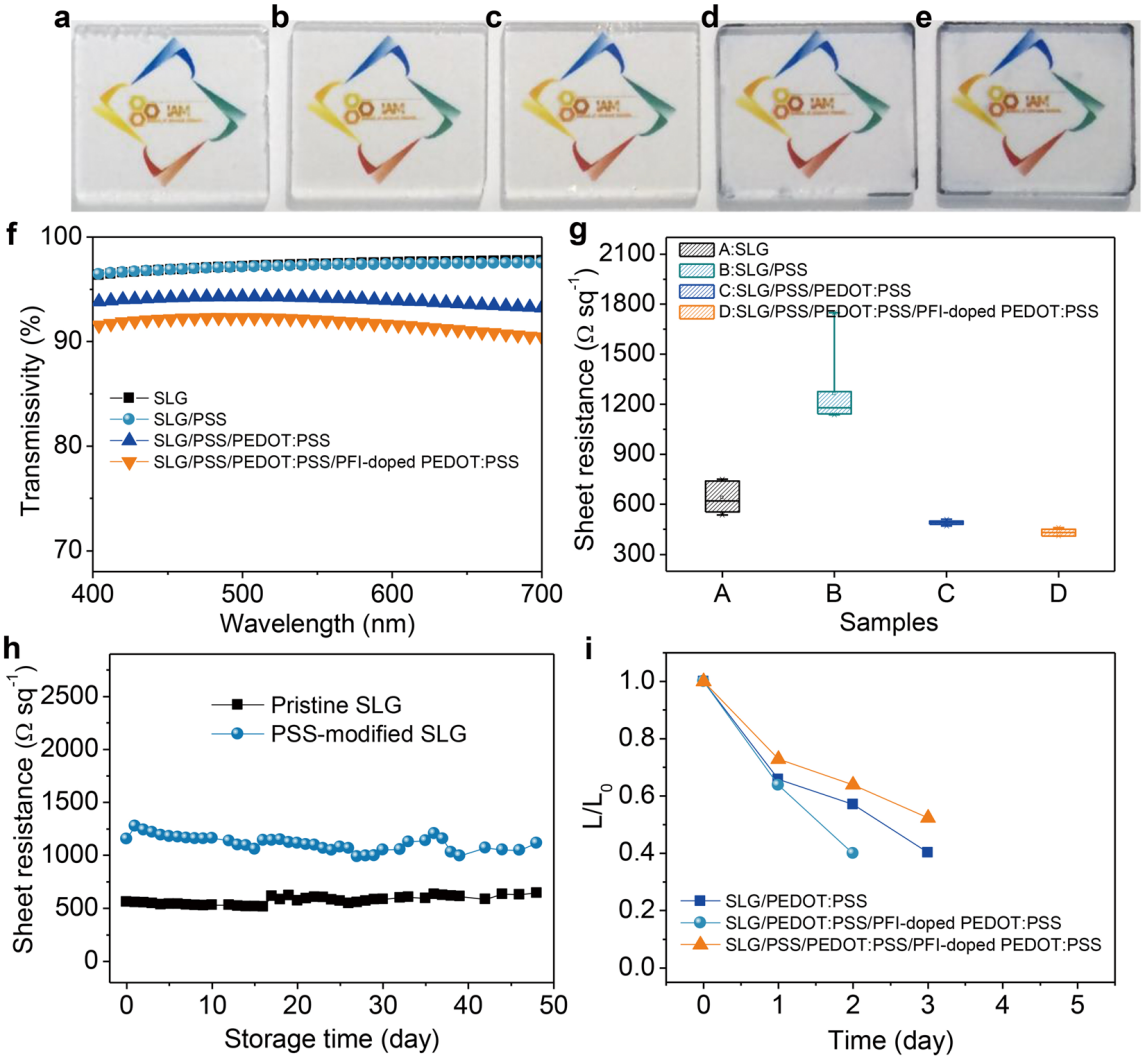
(**a**–**e**) Photographs of the glass substrate, monolayer graphene, graphene/PSS, graphene/PSS/PEDOT:PSS and graphene/PSS/PEDOT:PSS/PFI-doped PEDOT:PSS. (**f**) Transmittance curves of graphene, graphene/PSS, graphene/PSS/PEDOT:PSS and graphene/PSS/PEDOT:PSS/PFI-doped PEDOT:PSS films. All samples are prepared on quartz substrates. (**g**) Statistics on sheet resistance of pristine graphene (A), PSS-modified graphene (B), PEDOT:PSS-covered graphene/PSS (C) and PEDOT:PSS:PFI-coated graphene/PSS/PEDOT:PSS (D). (**h**) Sheet resistance of pristine graphene and PSS-modified graphene as a function of storage time. These graphene sheets are stored in air. (**i**) The relative luminance (L/L_0_) of devices *vs* time. The devices are aged by storing them without encapsulation in ambient air (25 °C and a relative humidity of 35%). The initial luminance (L_0_) of these devices is 1000 cd m^−2^.

The influence of PSS on graphene is further confirmed with analysis technique of Raman spectroscopy (Supplementary Fig. S1) and X-ray photoelectron spectroscopy (XPS, Fig. 2). Supplementary Fig. S1f–h reports statistical Raman shifts of over 20 data points for intrinsic and PSS-modified graphene samples. Compared with the intrinsic one, weak Raman shifts of the D, G and 2D band peaks are observed in the modified graphene with a low PSS concentration of 3 × 10^−6^ wt%. Moreover, the XPS analysis (Fig. 2) indicates that the characteristic C1s graphene peak shows no obvious shift towards lower binding energies and it rules out the possibility of doping^28^. The sheet resistance of up to 1,200 ± 200 Ω sq^−1^ (Fig. 3g) after PSS modification also demonstrates this point, which is different from the *p*-type doping with universal decrease in sheet resistance^1,2,29,30^. To further investigate possible influence of the PSS insulator on graphene’s interface energy level, the He Iα ultraviolet photoelectron spectroscopy (UPS) spectra of the intrinsic and the PSS-modified graphene are analyzed (Fig. 1d–g). Upon the coating of PSS, the photoemission onset (Fig. 1e) shifts towards a lower binding energy which corresponds to a higher Fermi level (E_F_) of 4.79 eV (4.51 eV for pristine graphene), as the energy level diagram drawn in Fig. 1h, from which a vacuum level (E_V_) bending of ~0.3 eV is observed at the graphene/PSS interface induced by interfacial dipoles and this level bending helps to reduce barrier for hole injection. The concept of using insulating materials for controlling the energy level has been commonly used in organic optoelectronic devices with Al electrodes^31–33^, but few reported in devices with graphene electrodes. Herein, the insulated polymer PSS reduces hole’s injection barrier and then improves device’s electroluminescent (EL) performances via dipole-induced E_V_ bending, instead of forming a *p*-type doping to graphene.

### Film stability

Notably, in contrast with a rapid recovery to high resistance state of pristine graphene for most chemical doping^1,24^, the PSS-modified SLG film in this work shows an excellent air-stability. As illustrated in Fig. 3h and Supplementary Fig. S2, by storing these graphene sheets in ambient air, the sheet resistance of the PSS-modified SLG film only exhibits a slight variation in the range of 10% with the storage time up to 30 days, which performs similar to that of the pristine graphene, indicating an excellent stability of PSS modification on graphene. With increasing measurement times, some local region is destroyed due to multiple contacts of graphene with probes of four-point probe instrument and this leads to a significant increase in sheet resistance of some points, as the statistics shown in Supplementary Fig. S2. In addition, the ultrathin PSS modification layer shows a negligible effect on the transmittance of the SLG film (PSS-coated SLG in Fig. 3c *vs* pure SLG in Fig. 3b) and a high transmissivity of 97.4% (at 550 nm) is maintained after PSS modification (Fig. 3f), which sufficiently restrains microcavity effect and is particularly beneficial to white light acquirement.

### Gradient hole injection layer

High sheet resistance of the PSS-modified SLG (1,200 ± 200 Ω sq^−1^) is compensated by the following coatings of PEDOT:PSS and PFI-doped PEDOT:PSS and this value decreases to 490 ± 20 and 437 ± 23 Ω sq^−1^ (Fig. 3g), respectively, which are comparative with or even slightly lower than those of pristine SLG. Obvious decrease in sheet resistance after the PEDOT:PSS coating is caused by the *p*-type doping of PEDOT:PSS to SLG, while its further decrease with an additional PFI-doped PEDOT:PSS layer is induced by some structural rearrangement of PEDOT:PSS into nanofibrils (Fig. 1b) due to the additive PFI doping^34^. With this PSS interfacial dipole layer and the PEDOT:PSS/PFI-doped PEDOT:PSS bilayer to form a gradient hole injection from 4.51 (pure SLG) to 5.48 eV (See the He I UPS spectra of graphene, PSS, PEDOT:PSS and PFI-doped PEDOT:PSS film samples in Fig. 1d–g), the injection barrier of holes greatly reduces from pure SLG to the hole transport layer 1,1-bis-(4-bis(4-methyl-phenyl)-amino-phenyl)-cyclohexane (TAPC) (with the highest occupied molecular orbital (HOMO) of 5.8 eV). With this gradient HIL (Fig. 1h), the turn-on voltage (*V*_on_) in white OLEDs is thus significantly decreased from 5 V (with PEDOT:PSS) and 4 V (with PEDOT:PSS/PFI-doped PEDOT:PSS bilayer) to 3 V.

### High-performance white OLEDs

**Figure 4:**
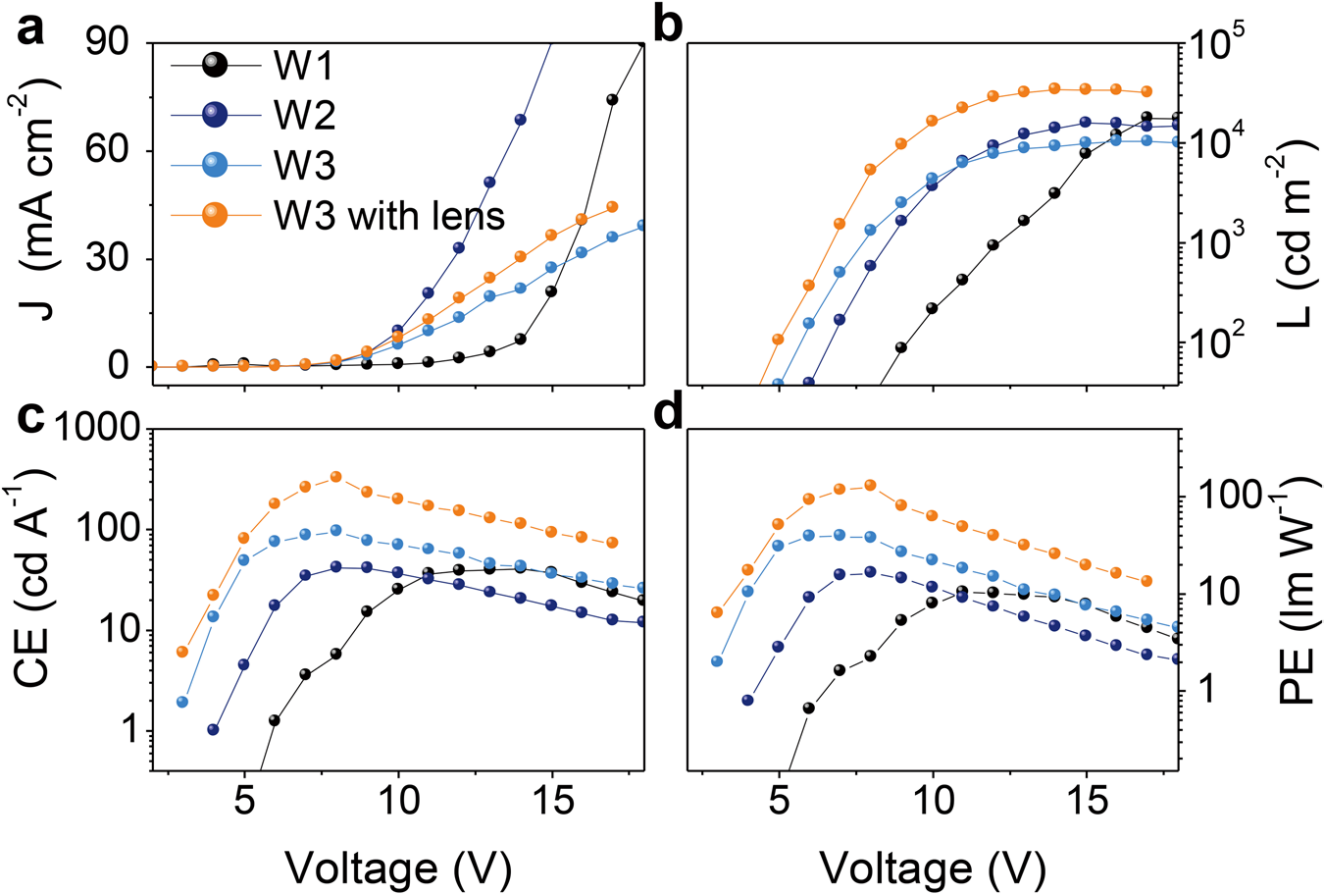
(**a**–**d**) The *J*-*V*, L, CE and PE curves of W1, W2 and W3, respectively. W1, W2 and W3 correspond to the HIL of pure PEDOT:PSS, the PEDOT:PSS/PFI-doped PEDOT:PSS and the PSS/PEDOT:PSS/PFI-doped PEDOT:PSS. Orange curves describe *J*-*V*, L, CE and PE characteristics of W3 with a high refractive index half ball lens on the substrate.

**Figure 5:**
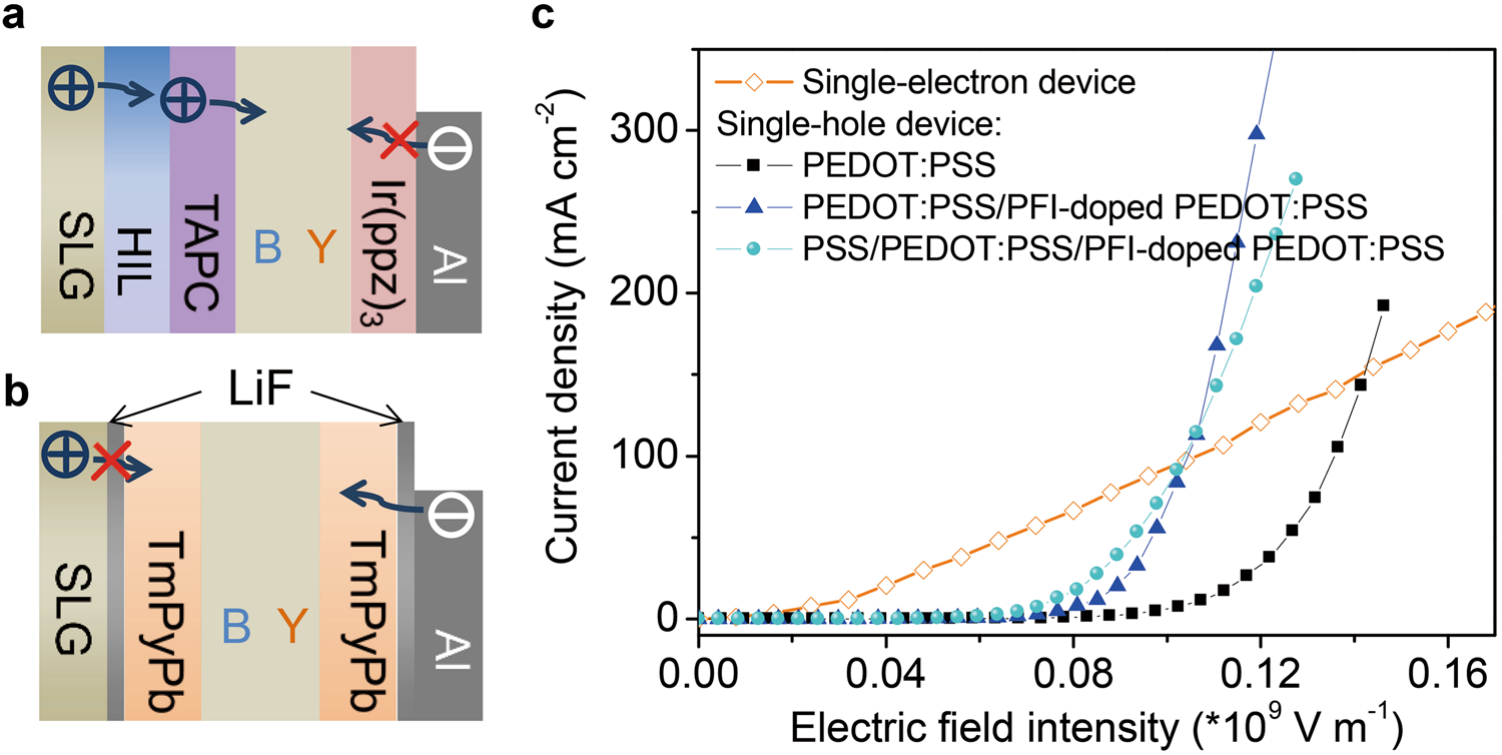
Schematic diagrams of (**a**) single-hole and (**b**) single-electron devices. (**c**) *J*-*V* characteristics of single-carrier devices. Note that single-hole devices were respectively fabricated with three different HILs of pure PEDOT:PSS, the PEDOT:PSS/PFI-doped PEDOT:PSS bilayer and the PSS/PEDOT:PSS/PFI-doped PEDOT:PSS trilayer.

As illustrated in Fig. 4a, compared with conventional white OLEDs with PEDOT:PSS as a HIL (W1), the injected current densities are remarkably enhanced in the counterparts with the PEDOT:PSS/PFI-doped PEDOT:PSS bilayer (W2) and the PSS/PEDOT:PSS/PFI-doped PEDOT:PSS trilayer (W3) as the HIL (detailed device structures for white emissions see device fabrication and characterization of Methods Section). Compared with W2, W3 exhibits a decrease in current density and an obvious increase in EL intensity, especially the luminance at low driving voltages. In order to explain this phenomenon, we design single-carrier devices with structures and fabrication process depicted in Methods Section. From the measured current density-voltage (*J*-*V*) curves of single-hole and single-electron devices in Fig. 5, we notice that the injected electron current is far larger than the hole counterpart in W1 only using PEDOT:PSS as HIL, and the hole injection is significantly improved with the use of PFI and PSS. In the case of PSS-modified device, the hole current is further improved at an electric field intensity of no more than 10^8^ V m^−1^ (corresponding to a driving bias of less than 12 V), indicating a beneficial improvement of the gradient HIL to hole injection. However, we find a larger *J* in W2 than that in W3, as illustrated in Fig. 4a, which is in contrast with the trend in single-hole devices. And this result suggests a possible large leakage current in W2 due to a weak blocking effect of perfluorinated ionomers PFI on electrons, which does not contribute to performance improvement of white devices, as the luminance (L), current efficiency (CE) and power efficiency (PE) curves shown in Fig. 4b–d and the external quantum efficiency (EQE) curves illustrated in Supplementary Fig. S3a. In contrast, W3 provides a large injection barrier for electrons towards anode due to a wide band gap of the insulating PSS. Thus, W3 shows a maximum CE, PE and EQE of 95.9 cd A^−1^ (8 V), 39.3 lm W^−1^ (7 V) and 29.2% (8 V), exhibiting 2.29, 2.40 and 1.95-fold enhancements compared with 41.9 cd A^−1^ (8 V), 16.4 lm W^−1^ (8 V) and 15.0% (8 V) with PEDOT:PSS/PFI-doped PEDOT:PSS in W2. It is worth mentioning that L in W3 exhibits a 6.45-fold enhancement at 4 V and then decreases to 1.18-fold when the driving voltage increases to 10 V. Note that the maximum L in W3 (13,420 cd m^−2^ @ 17 V) shows a slight decrease compared with that in W2 (15,780 cd m^−2^ @ 15 V) due to a relatively low current. When a high refractive index half ball lens (*n* = 1.922) is covered on the light output side of the W3′s glass substrate, very high L, CE, PE and EQE of 34,380 cd m^−2^ (14 V), 326.5 cd A^−1^ (8 V), 128.2 lm W^−1^ (8 V) and 99.5% (8 V) are achieved in W4, amounting to a ~3.7, 3.4, 3.3 and 3.4-fold enhancements over luminance, CE, PE and EQE in W3. This significant enhancement is due to an improved light outcoupling with a high refractive index hemispherical lens. We must clarify that the optimal EL efficiency in this work is achieved at a high luminance of 5,270 cd m^−2^, different from reported results in literature, which is usually obtained at a low brightness and significantly decreases with driving bias. To the best of our knowledge, up to now, the maximum luminous efficiency of white OLEDs is ~130 cd A^−1^ and 123.4 lm W^−1^ at a luminance of 1,000 cd m^−2^, reported by Tang *et al*.^35,36^, and it decreases to 106.5 lm W^−1^ when L is up to 5,000 cd m^−2^. Our results demonstrate a potential of graphene anodes for applications in white OLED lighting. In addition to synthesizing high quality graphene and developing efficient transfer method, graphene modification and device structure design also play an important role in fabricating high efficient OLEDs. The white emission of W3 in this paper exhibits a stable pure white color with a correlated color temperature of 4,200 ± 100 K and a Commission International de L’Eclairage coordinate (CIE) change within (0.029, 0.014) at a luminance range of 300–10,000 cd m^−2^ (See the EL spectra in Supplementary Fig. S3d and data collection in Table 1).

**Table 1:** Summarized device performances.

Device		*V*_on_ [V]^a^	L_max_@*V* [cd m^−2^ @V]^b^	CE_max_; PE_max_ [cd A^−1^; lm W^−1^]^c^	CIE (x, y) @300 [cd m^−2^]^d^	CIE (x, y) @5,000 [cd m^−2^]	CIE (x, y) @10,000 [cd m^−2^]
White	W1	5.0	17,630@17	40.8; 10.4	(0.333, 0.442)	(0.350, 0.450)	(0.353, 0.452)
	W2	4.0	15,780@15	41.9; 16.4	(0.339, 0.440)	(0.361, 0.450)	(0.363, 0.452)
	W3	3.0	13,420@17	95.9; 39.3	(0.337, 0.440)	(0.365, 0.453)	(0.366, 0.454)
	W3 with lens	2.8	34,380@14	326.5; 128.2	(0.340, 0.441)	(0.366, 0.453)	(0.365, 0.454)
Blue	B3	3.7	6,477@15	73.8; 26.9	(0.161, 0.354)	(0.167, 0.367)	
	B3 with lens	3.6	13,283@15	201.9; 76.1	(0.161, 0.353)	(0.162, 0.355)	

^a^*V*_on_: turn-on voltage; ^b^L_max_: the maximum luminance; ^c^CE_max_, PE_max_: the maximum current and power efficiency; ^d^CIE (x, y): Commission International de L’Eclairage coordinate.

### High-performance blue OLEDs

**Figure 6:**
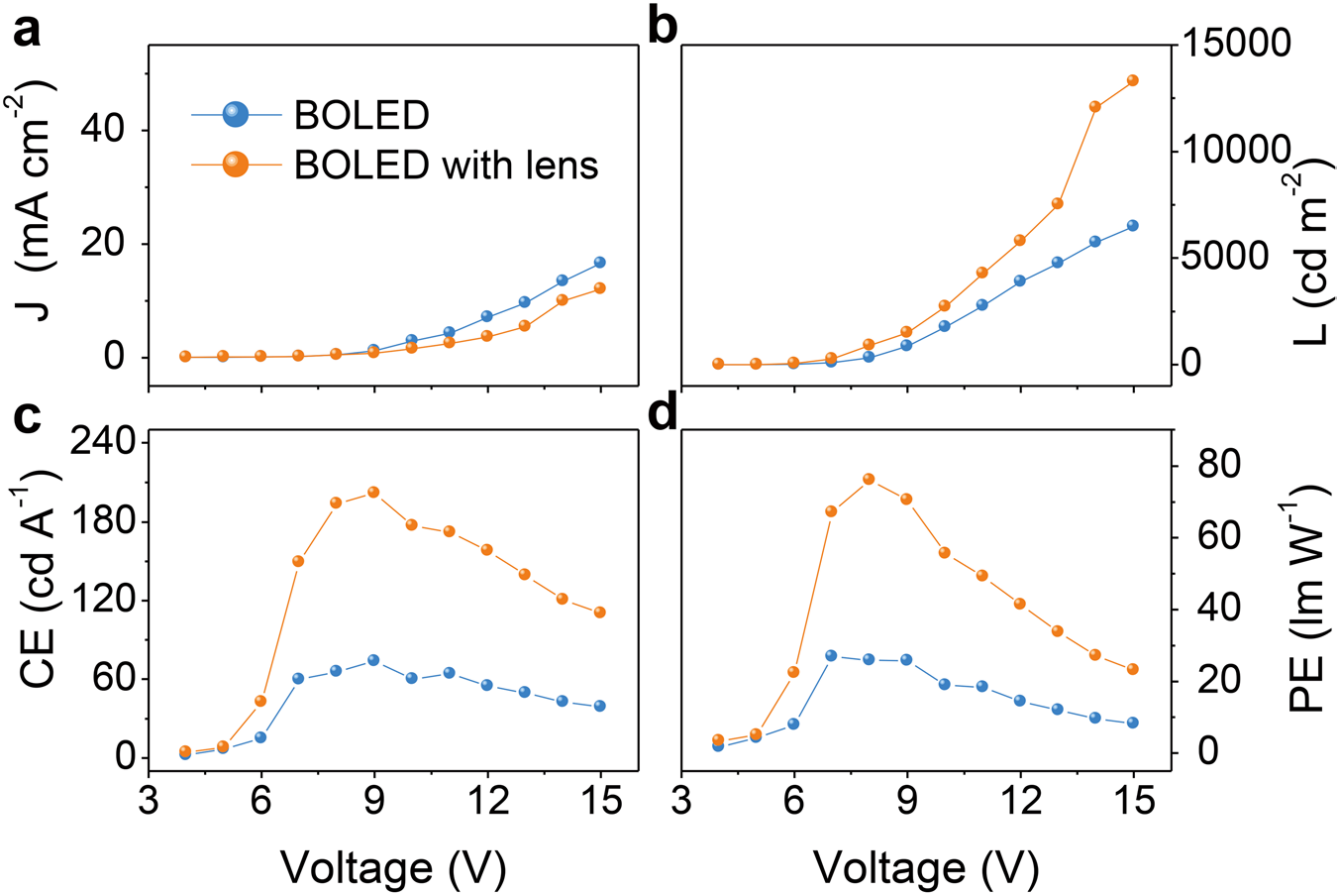
(**a**) *J*-*V*, (**b**) L, (**c**) CE and (**d**) PE curves of B3 with and without half ball lens.

Totally different from a microcavity resonance-enhanced approach to enhance EL efficiency^26^, the method in this work is irrelative to the emission color and suitable for fabrication of both monochromatic and white OLEDs. To verify this, blue emission OLEDs are manufactured with our interfacial modification layer and gradient HIL structure, with device configuration described in Methods Section. Similar enhancement effects are observed in these blue emission devices with iridium(III)bis[4,6-(di-fluorophenyl)-pyridinato-N,C^2′^]picolinate (FIrpic) phosphors. The maximum CE, PE and EQE achieve 73.8 cd A^−1^ (at 866 cd m^−2^), 26.9 lm W^−1^ (at 97 cd m^−2^), 16.5% (at 866 cd m^−2^) and 201.9 cd A^−1^ (at 1,494 cd m^−2^), 76.1 lm W^−1^ (at 896 cd m^−2^), 45.2% (at 1,494 cd m^−2^) for blue emissions without and with a high refractive index hemispherical lens, respectively (Fig. 6, Supplementary Fig. S4a and Table 1). The blue emission also shows a negligible alteration of spectra at a large bias range of 4–15 V regardless of high refractive index hemispherical lens (Supplementary Fig. S4b and Table 1).

### Device stability

Lifetime of blue OLEDs is measured to observe device stability after PSS modification. Note that the devices in this work are aged by storing them without encapsulation in ambient air (25 °C and a relative humidity of 35%) and are measured once each day. Tested for 72 hours, the devices with the PSS/PEDOT:PSS/PFI-doped PEDOT:PSS gradient HIL remain ~52% of an initial luminance (1000 cd m^−2^), while the luminance decreases to ~40% in the control one with the PEDOT:PSS HIL only (Fig. 3i). Above results demonstrate that the PSS-modified devices show a slight better stability than the control one without any modification, which is mainly due to a good air-stability of the PSS-modified SLG film (Fig. 3h) and a reduction of leakage current inside the OLEDs with the use of PSS^37^.

## Discussion

Graphene as a novel electrode material with excellent transparency, conductivity and mechanical compliance, still faces enormous obstacles for successful applications in flexible OLEDs. These challenges include graphene’s surface modification with long-term stability, large area transfer technology of graphene, and encapsulation of OLEDs based on graphene electrodes. This work presents a stable interface modification method to graphene with an insulated PSS, with the formation of a vacuum energy level bending due to the interfacial dipole at the interface of graphene and HIL. By combining this interface modification technique with a gradient hole injection layer, highly efficient blue and white OLEDs with current (power and external quantum) efficiencies of 201.9 cd A^−1^ (76.1 lm W^−1^ and 45.2%) and 326.5 cd A^−1^ (128.2 lm W^−1^ and 99.5%) are achieved, respectively. In addition to high efficiency, the white emission also exhibits excellent color temperature and color stability, and all these parameters are sufficient for general display and lighting applications. The understanding gained from this study is expected to allow for the construction of high-performance optoelectronic devices that could find new applications in the fields of perovskite light-emitting diodes, solar cells, thin-film transistors, and potentially many others employing graphene-based electrodes.

## Methods

### Graphene transfer

The as-used SLG was synthesized with a chemical vapor deposition process using copper foils as substrates. Transfer method of SLG was referred to our previous work^38^. It is worth noting that the SLG was washed with hot acetone (60 °C) to sufficiently remove surface poly(methyl methacrylate) residue and this step was repeated at least three times, which can eliminate some abnormal electrical phenomena^39^.

### Film characterization

The transmittance and sheet resistance of as-transferred SLG were measured with an ultraviolet-visible spectrophotometer (Shimadzu, UV-3600) and a four-point probe (RTS-9, China). The components of graphene were analyzed with Raman spectrometer (Reinshaw, InVia). The work function of graphene and the E_F_ of each HIL were measured with UPS (Kratos Axis Ultra^α^ ultrahigh vacuum surface analysis system) and these values were calculated by using the photoemission onset to subtract the excitation energy of 21.22 eV. The C1s spectra of graphene sheets with and without PSS modification were measured with XPS (Kratos Axis Supra). Film thicknesses except an ultrathin PSS were directly measured with a Stylus Profiler (Bruker, DektakXT). The thickness for the ultrathin PSS interfacial modification layer was inferred with an equation of $I/{I}_{0}={e}^{-\alpha x}$ following the steps below: First, the thicknesses (*x*) and transmissivities (*I/I*_*0*_) of several groups of different PSS film thicknesses were measured with a Stylus Profiler and an ultraviolet-visible spectrophotometer to extract the PSS film’s absorption coefficient (α). Then, the transmissivity of the PSS ultrathin film used in this work (spin-coating the PSS aqueous solution with 3 × 10^−6^ wt% concentration with a rotation ratio of 3,000 rpm for 60 s to form the ultrathin film) was measured. The thickness of the PSS ultrathin film was eventually calculated with above **α** and transmissivity, with an average value of 2.1 ± 0.2 nm.

### Device fabrication and characterization

The as-transferred SLG sheets were dried on a hot plate at 120 °C for 30 min before device fabrication. Afterwards, the graphene-covered substrates were treated with UV O_3_ for 6 min and then transferred onto a spin-coater to spin-coat the PEDOT:PSS (W1), the PEDOT:PSS/PFI-doped PEDOT:PSS bilayer (W2) and the PSS/PEDOT:PSS/PFI-doped PEDOT:PSS trilayer (W3) as a HIL. Here, PEDOT:PSS was brought from Heraeus (Clevios™ P VP AI 4083) with a resistivity of 500–5,000 Ω cm. After a dry process at 120 °C for 30 min, the substrates were loaded into a vacuum chamber to thermally deposit the hole transport layer TAPC, the emitting layer (EML), the electron transport layer 1,3,5-tri(m-pyrid-3-yl-phenyl)benzene (TmPyPb, 50 nm), the electron injection layer LiF (0.5 nm) and the cathode Al (100 nm) in sequence, forming final devices with an emitting area of 0.05 cm^−2^. The EML in white OLEDs is composed of a 19-nm blue light layer and a 1-nm orange-yellow ultrathin one *via* doping 8 wt% FIrpic and 10 wt% Iridium(III)bis(4-phenylthieno[3,2-*c*]pyridinato-*N*,C^2′^)acetylacetonate (PO-01) phosphorescent guests into the *N*,*N*’-dicarbazolyl-3,5-benzene (mCP) host. While for the blue OLEDs, the EML is comprised of 30 nm FIrpic (8 wt%)-doped mCP. Blue emission with the trilayer HIL of PSS/PEDOT:PSS/PFI-doped PEDOT:PSS was denoted as B3, and the parameters of B3 with and without lens were summarized in Table 1. All inorganic/organic layers were thermally deposited in a high-vacuum system with a pressure of less than 5 × 10^−4^ Pa and deposition rates of 0.5–10 Å s^−1^ (5–10 Å s^−1^ for Al and 0.5–1 Å s^−1^ for other inorganic/organic layers).

Single-hole and single-electron devices with the device configurations of SLG/HIL/TAPC (40 nm)/mCP: 8 wt% FIrpic (19 nm)/mCP: 10 wt% PO-01 (1 nm)/tris(phenypyrazole)iridium (Ir(ppz)_3_, 100 nm)/Al (100 nm) and SLG/LiF (0.5 nm)/TmPyPb (50 nm)/mCP: 8 wt% FIrpic (19 nm)/mCP: 10 wt% PO-01 (1 nm)/TmPyPb (50 nm)/LiF (0.5 nm)/Al (100 nm) were fabricated *via* spin-coating or thermal deposition process similar to the aforementioned OLEDs. Here, the HILs are PEDOT:PSS (1,500 rpm for 60 s, 45 nm), PEDOT:PSS (1,500 rpm for 60 s, 45 nm)/PFI-doped PEDOT:PSS (3,000 rpm for 60 s, 30 nm) and PSS (3 × 10^−6^ wt%, 3,000 rpm for 60 s)/PEDOT:PSS (1,500 rpm for 60 s, 45 nm)/PFI-doped PEDOT:PSS (3,000 rpm for 60 s, 30 nm), which corresponds to devices W1, W2 and W3, respectively. Current density *vs* electric field intensity characteristics were drawn to ensure that current densities were compared under the same electric field intensity (Fig. 5). Note that the doping ratio of PFI to PEDOT:PSS was 1:2 in volume for all devices.

The EL characteristics were measured with a PR655 spectrometer and a Keithley 2400 programmable voltage-current source. The light output was improved by covering 5 mm diameter half ball lens with a refractive index of 1.922 onto the glass substrates. All devices including blue OLEDs for lifetime measurements were directly operated at room temperature in ambient air without encapsulation.

### Data availability

All data generated or analyzed during this study are available from the corresponding author.

## Electronic supplementary material

Supplementary information
