# Supplementary material for: Interfacial engineering of graphene for highly efficient blue and white organic light-emitting devices
Source: Sci Rep. 2018 May 25;8:8155. doi: 10.1038/s41598-018-26464-8 (PMC5970198; doi:10.1038/s41598-018-26464-8)
Supplement: Supplementary file 1 — Supplementary information [file 41598_2018_26464_MOESM1_ESM.pdf]

Supplementary Information

Interfacial engineering of graphene for highly efficient blue and white organic light-emitting devices

Shufen Chen[1], Qin Zhang[1], Wenjuan Shang[1], Lihui Liu[1], Hongtao Yu[1], Shuai Zhang[1], Lingling Deng[1], Min Wang[1], Minghao Wang[1], Xue Li[2], Baoxiu Mi[1] & Wei Huang[1,3]

[1]Key Laboratory for Organic Electronics and Information Displays & Jiangsu Key Laboratory for Biosensors, Institute of Advanced Materials (IAM), Jiangsu National Synergetic Innovation Center for Advanced Materials (SICAM), Nanjing University of Posts & Telecommunications, 9 Wenyuan Road, Nanjing 210023, China.

[2]Mechanical Engineering Institute, Nanjing Institute of Technology, Nanjing 211167, China.

[3]Institute of Flexible Electronics (SIFE), Northwestern Polytechnical University (NPU), 127 West Youyi Road, Xi'an 710072, Shaanxi, China.

Shufen Chen, Qin Zhang, Wenjuan Shang and Lihui Liu contributed equally to this work.

Correspondence and requests for materials should be addressed to S.C. (email: iamsfchen@njupt.edu.cn) or W.H. (email: wei-huang@njtech.edu.cn)

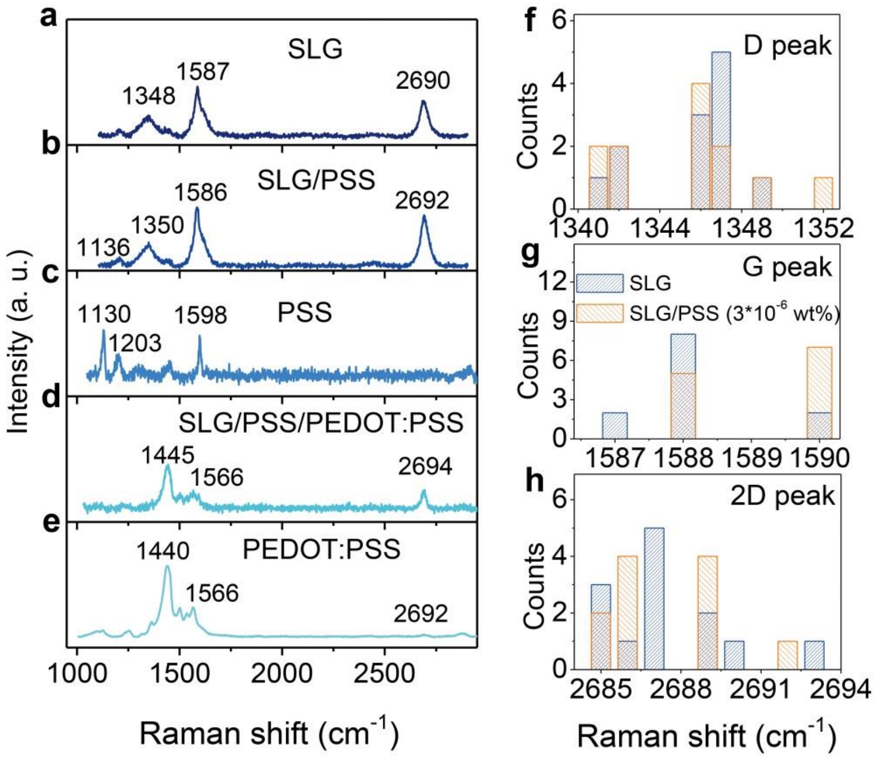

Figure S1. Raman spectra of (a) graphene, (b) PSS-modified graphene, (c) PSS, (d) PEDOT:PSS-coated graphene/PSS and (e) PEDOT:PSS. (f-h) Statistics on Raman shift of the D, G, and 2D characteristic peaks for pristine and PSS-modified graphene sheets. Here, the PSS concentration that modifies the monolayer graphene is 3×10^{-6} wt%, with which an optimal device performance is obtained.

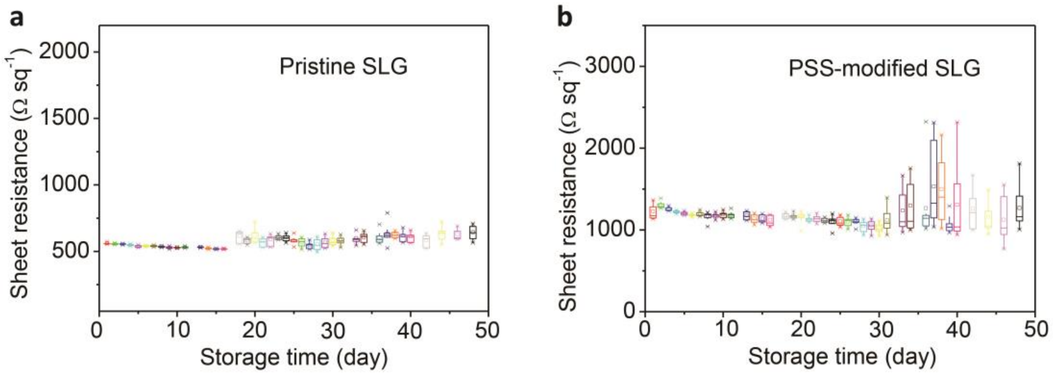

Figure S2. Statistics on sheet resistance of (a) pristine and (b) PSS-modified graphene sheets.

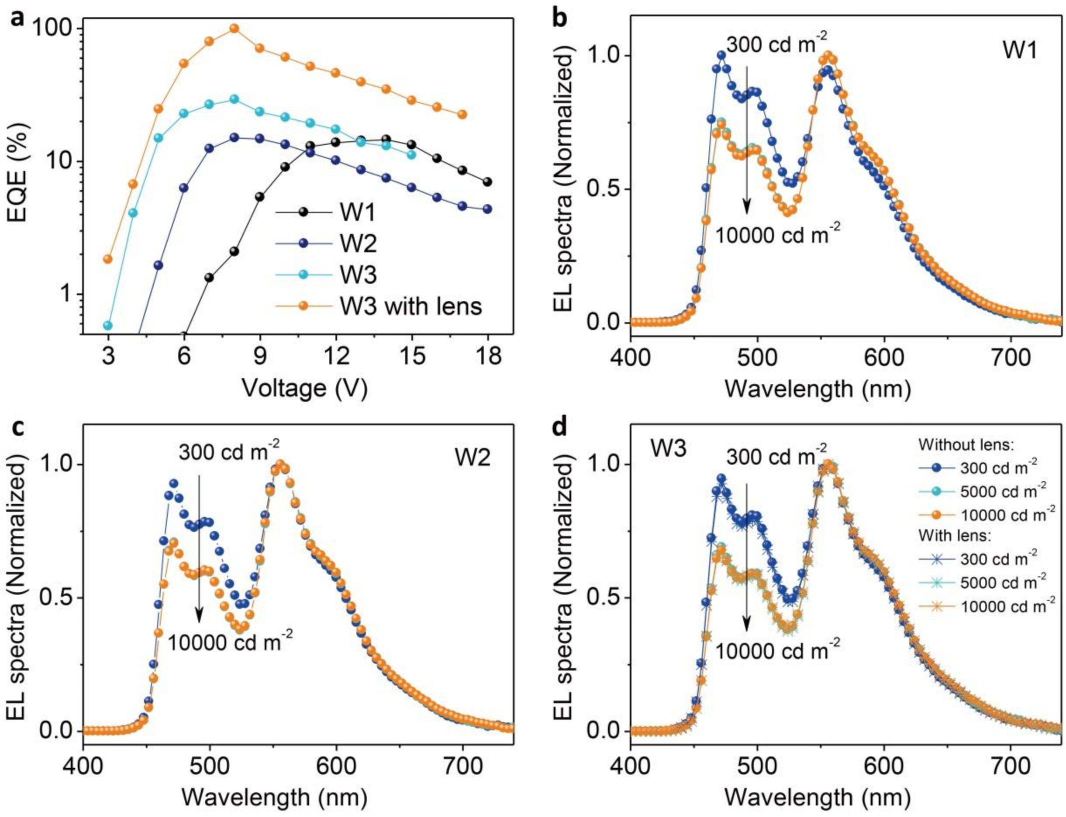

Figure S3. (a) The EQE curves of W1, W2 and W3 (with and without half ball lens) and alteration of the EL spectra for (b) W1, (c) W2 and (d) W3 (with and without half ball lens) with brightness.

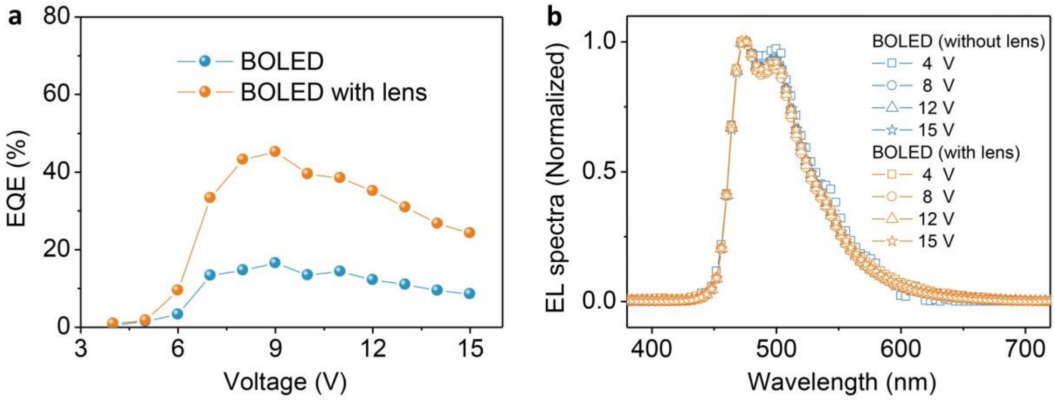

Figure S4. (a) The EQE curves of B3 with and without half ball lens, and (b) alteration of the EL spectra for B3 (with and without half ball lens) with driven bias.
